# Draft Genome Sequence of Pectobacterium carotovorum subsp. carotovorum ATCC 39048, a Carbapenem-Producing Phytopathogen

**DOI:** 10.1128/MRA.00825-18

**Published:** 2018-08-09

**Authors:** Rita E. Monson, Katherine Jones, George P. C. Salmond

**Affiliations:** a Department of Biochemistry, University of Cambridge, Cambridge, United Kingdom; University of California, Riverside

## Abstract

Pectobacterium carotovorum subsp. carotovorum ATCC 39048 was originally isolated in the 1980s and studied because it produced the β-lactam antibiotic 1-carbapen-2-em-3-carboxylic acid. The draft genome for this strain was 4,637,928 bp with a G+C content of 51.98%. The genome contained the carbapenem biosynthetic cluster, genes encoding plant virulence determinants, and a single metallo-β-lactamase.

## Abstract (precis)

Pectobacterium carotovorum subsp. carotovorum ATCC 39048 was originally isolated in the 1980s and studied because it produced the β-lactam antibiotic 1-carbapen-2-em-3-carboxylic acid.

## ANNOUNCEMENT

Pectobacterium carotovorum subsp. carotovorum ATCC 39048 (Pcc39048) is a Gram-negative member of the Pectobacteriaceae family. It is a pathogen of potato, causing soft rot in tubers. It is of pharmaceutical interest, as it produces the β-lactam antibiotic 1-carbapen-2-em-3-carboxylic acid (a carbapenem). Here, we report the draft genome sequence of Pcc39048.

The draft genome was generated using Pcc39048 genomic DNA isolated from an overnight culture grown in lysogeny (Lennox) broth (10 g/L tryptone, 5 g/L yeast extract, 5 g/L NaCl), extracted using phenol chloroform, and recovered by ethanol precipitation (1). The sequencing was undertaken using the Illumina HiSeq platform with 2 × 250-bp reads. The reads were trimmed using Trimmomatic (2) and assembled with SPAdes using the default settings (3). Read quality and assembly were assessed using BWA-MEM (4). The genome comprised 40 contigs with a mean coverage of 127.20× and an *N*_50_ of 220,041 bp. The genome was annotated using the NCBI Prokaryotic Genome Annotation Pipeline. The draft genome contained 4,637,928 bp with a 51.98% G+C content. We identified 4,208 open reading frames, 68 tRNAs, 12 rRNAs, and 1 transfer-messenger RNA. Two CRISPR arrays surrounding sequences encoding four Cas proteins (Csy1 through Csy4) and two putative transposases were also identified. The CRISPR array organization was similar to that of P. atrosepticum SCRI1043. However, the Pcc39048 draft genome contained two CRISPR arrays rather than three, and the P. atrosepticum CRISPR region does not contain the two transposases (5).

Pcc39048 has been studied since the 1980s due to its production of a simple carbapenem antibiotic (6). The biosynthetic cluster for carbapenem production has been identified (7) and was also present within this draft genome. In addition to intrinsic carbapenem resistance genes (*carF* and *carG*), the genome also encoded a metallo-β-lactamase unlinked to the carbapenem antibiotic locus. In Pcc39048, we identified an *N*-acyl-homoserine lactone (AHL) quorum-sensing system and a single AHL synthase, encoded by *expI*, that was convergently transcribed with a LuxR-type transcriptional regulator gene, *expR*. The genome also contained *virR*, which encodes another LuxR-type regulator in many members of the family Pectobacteriaceae (8). Upstream of the carbapenem production operon, a third LuxR-family regulator of carbapenem production, *carR*, was identified.

Secreted proteins are important for Pcc39048 plant infection, and pectinases and cellulases were both identified in the draft genome (9). We also identified the necrosis-inducing virulence gene (*nip*) encoding a homologue of a fungal and oomycete elicitor, Nep1 (necrosis and ethylene-inducing peptide [10]). The genome sequence of Pcc39048 possessed the hypersensitive response and pathogenicity hairpin gene *hrpN*, which is involved in eliciting the hypersensitive response in resistant hosts and nonhost plants (11). Finally, we identified an orthologue of *svx* encoding a virulence determinant secreted via the type II (out) nanomachine (12).

The draft genome sequence of Pcc39048 described here identified the carbapenem biosynthetic cluster, a quorum-sensing system (comprising a single acyl homoserine lactone synthase and three partner transcriptional regulators of the LuxR-family), and a CRISPR-*cas* system. We hope that this genome is a useful resource for researchers interested in enterobacterial plant pathology and antibiotic production.

### Data availability.

The draft genome sequence reported here has been deposited at GenBank under the accession no. QHMC00000000. The version described here is the first version, QHMC01000000.
